# mTOR pathway inhibition alters proliferation as well as differentiation of neural stem cells

**DOI:** 10.3389/fncel.2024.1298182

**Published:** 2024-05-15

**Authors:** Nataliya Romanyuk, Kristyna Sintakova, Ivan Arzhanov, Martin Horak, Chirag Gandhi, Meena Jhanwar-Uniyal, Pavla Jendelova

**Affiliations:** ^1^Department of Neuroregeneration, Institute of Experimental Medicine of the Czech Academy of Sciences, Prague, Czechia; ^2^Department of Neuroscience, Second Faculty of Medicine, Charles University, Prague, Czechia; ^3^Department of Neurochemistry, Institute of Experimental Medicine of the Czech Academy of Sciences, Prague, Czechia; ^4^Department of Neurosurgery, New York Medical College, Valhalla, NY, United States

**Keywords:** neural stem cells, mTOR, proliferation, differentiation, cytoskeletal proteins

## Abstract

**Introduction:**

Neural stem cells (NSCs) are essential for both embryonic development and adult neurogenesis, and their dysregulation causes a number of neurodevelopmental disorders, such as epilepsy and autism spectrum disorders. NSC proliferation and differentiation in the developing brain is a complex process controlled by various intrinsic and extrinsic stimuli. The mammalian target of rapamycin (mTOR) regulates proliferation and differentiation, among other cellular functions, and disruption in the mTOR pathway can lead to severe nervous system development deficits. In this study, we investigated the effect of inhibition of the mTOR pathway by rapamycin (Rapa) on NSC proliferation and differentiation.

**Methods:**

The NSC cultures were treated with Rapa for 1, 2, 6, 24, and 48 h. The effect on cellular functions was assessed by immunofluorescence staining, western blotting, and proliferation/metabolic assays.

**Results:**

mTOR inhibition suppressed NSC proliferation/metabolic activity as well as S-Phase entry by as early as 1 h of Rapa treatment and this effect persisted up to 48 h of Rapa treatment. In a separate experiment, NSCs were differentiated for 2 weeks after treatment with Rapa for 24 or 48 h. Regarding the effect on neuronal and glial differentiation (2 weeks post-treatment), this was suppressed in NSCs deficient in mTOR signaling, as evidenced by downregulated expression of NeuN, MAP2, and GFAP. We assume that the prolonged effect of mTOR inhibition is realized due to the effect on cytoskeletal proteins.

**Discussion:**

Here, we demonstrate for the first time that the mTOR pathway not only regulates NSC proliferation but also plays an important role in NSC differentiation into both neuronal and glial lineages.

## 1 Introduction

Neural stem cells (NSCs) and their ability to differentiate into various types of neurons are an essential basis of neurogenesis, which is crucial not only during embryonic development but also during adulthood. The transition of NSCs to mature functional neurons is tightly regulated by multiple external and internal signals to ensure that the NSCs divide, differentiate, migrate, and integrate properly. One of the main regulators of these processes is the mammalian target of rapamycin (mTOR), which can converge multiple external signals. Several studies have suggested that mTOR is involved in many aspects of neurogenesis (Kassai et al., 2014; Lee et al., 2016; Mahoney et al., 2016; Mirzaa et al., 2016).

mTOR constitutes the catalytic subunit of two functionally distinct protein complexes, mTOR Complex 1 (mTORC1), sensitive to the allosteric inhibitor rapamycin (Rapa), and mTOR Complex 2 (mTORC2), which is insensitive to the Rapa treatment. mTOR complexes are part of the key intracellular signaling pathway of PI3K/Akt/mTOR. mTORC1 controls cell growth in terms of time, i.e., when the cell grows, whereas mTORC2 controls cell growth in terms of space, i.e., where the cell grows (Saxton and Sabatini, 2017). This is done by influencing the processes related to cytoskeletal remodeling, which determines the cell shape.

Following neuronal commitment, the rounded neuronal precursors form membrane sprouts that later develop into neurites and extend during neuronal differentiation (neurite outgrowth). The extending neurites form branches (neurite branching) that lead to axon collaterals or dendritic arbors. The components of the cytoskeleton not only control cell morphology but also form scaffolds for organelle transport and regulate growth cone motility and axon guidance. The eukaryotic cytoskeleton consists of filamentous proteins belonging to three major families of elements: (1) microtubules or MTs (25 nm of diameter, composed of dimers of α and β-tubulin), (2) actin filaments or AF (6 nm of diameter) and (3) intermediate filaments or IF (10 nm of diameter) (Compagnucci et al., 2016). Here we studied the MT component βIII-tubulin, microtubule-associated protein 2 (MAP2) and several IF components: neurofilament medium (NF-M) and heavy chain (NF-H) and glial fibrillary acidic protein (GFAP). We focused on the effect of mTORC1 inhibition on changes in their levels during neuronal and glial differentiation.

Both complexes respond to the presence of different signals such as growth factors, cell energy and oxidative stress. Cell growth is then adequately modulated in response to these signals (Wullschleger et al., 2006; Huang and Fingar, 2014). An aberrant mTOR pathway can lead to a severe deficit in nervous system development, including tumors, autism, and seizures (Saxton and Sabatini, 2017). Furthermore, mTORC1 is essential for the proliferation and survival of NSCs. Reduction of its activity causes a decrease in the population of neural progenitors. In addition, mTORC1 inhibition negatively affects the population of neurons generated postnatally in the subventricular zone (SVZ) while hyperactivation of this complex leads to accelerated differentiation of NSCs, thereby leading to their depletion (Hartman et al., 2013; Switon et al., 2017). This observation is supported by a previous finding which showed a strong effect of hyperactivated mTORC1 on the gradual loss of NSCs (Mahoney et al., 2016). In fact, Mahoney et al. (2016) demonstrated that mTORC1 activation induced terminal differentiation of NSCs in the neonatal SVZ but questioned its effect on proliferation. This conception is further confirmed by studies showing that the inhibition of mTOR by Rapa reduced neural differentiation without affecting proliferation (Lee et al., 2016). Insulin had a supportive effect on neural differentiation via the PI3K/Akt/mTOR signaling pathway. The level of phosphorylated mTOR was increased in the PI3K/Akt dependent neural progenitor differentiation pathway, which was induced by insulin (Chinta and Andersen, 2005; Han et al., 2008; Lee et al., 2016). Activation of mTOR by suppressing its negative regulators PTEN or TSC1 has been shown to promote axonal regeneration (Julien et al., 2010). In addition to its role in NSCs differentiation, the mTOR pathway is also involved in the process of dendrite formation, which is essential for proper signal transduction between neurons (Skalecka et al., 2016). The growing evidence for the involvement of the mTOR pathway in neurogenesis and its influence on NSC function does not leave unnoticed its central role in the above-mentioned processes. However, the results of several studies are contradictory and the mechanisms caused by the effect of mTOR on NSC differentiation need further elucidation.

The aim of this study was to determine the effect of mTOR pathway inhibition on NSC differentiation. We evaluated the effect of Rapa on the proliferation and differentiation of neural progenitors derived from induced pluripotent stem cells (iPSC-NPs). The results presented here elucidate the biochemical alterations that occur during NSC differentiation after mTOR inhibition.

## 2 Materials and methods

### 2.1 Cell culture

All experiments used neural precursors differentiated from human-induced pluripotent (iPSC-NPs) cells derived from fetal lung fibroblast line (iMR 90; ATCC, Manassas, VA, United States). The cells were cultured in T75 flasks (Nunc, Thermo Fisher Scientific, Roskilde, Denmark) coated with poly-L-ornithine (Sigma-Aldrich, St. Louis, MO, United States) and laminin (Sigma-Aldrich, St. Louis, MO, United States) in a CO_2_ incubator (MCO-170AICUVH-PE, Panasonic, Osaka, Japan) at 37°C. The medium for neural stem cells (NSC) consisted of: DMEM: F12 and neurobasal medium (1:1), supplements B27 (1:50) and N2 (1:100), a mixture of penicillin and streptomycin antibiotics (1: 200) (all from Thermo Fisher Scientific, Waltham, MA, United States), EGF (10 ng/mL), bFGF (10 ng/mL) and BDNF (10 ng/mL) (all from Pepro Tech, London, United Kingdom), antibiotics primocin (1:500) (Invivo Gen, San Diego, CA, United States). This medium was replaced every other day.

**Figure 1:**
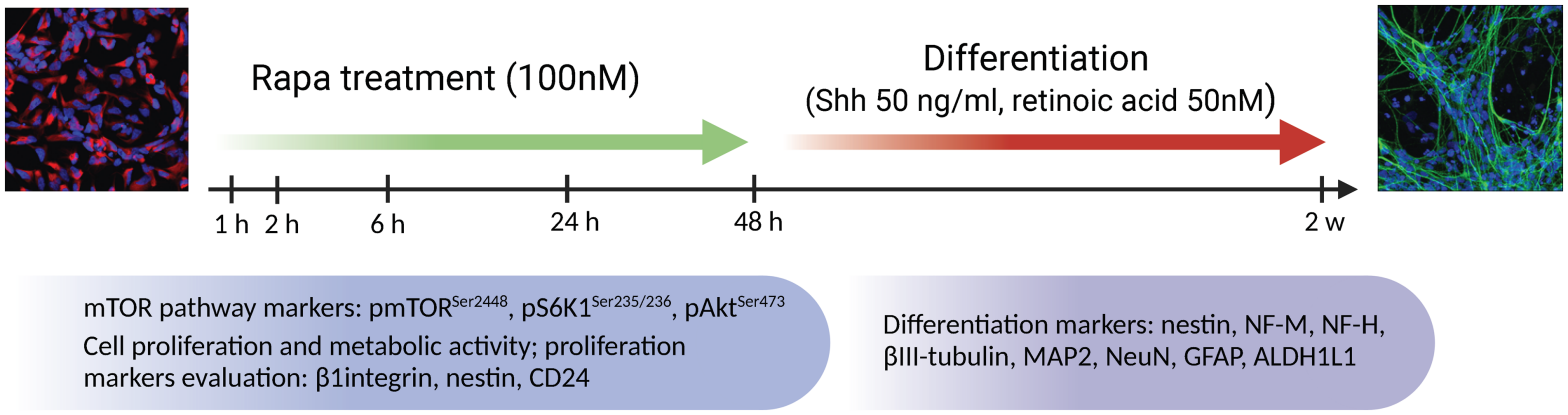
Treatment algorithm of NSC culture with the mTOR inhibitor rapamycin. NSCs were treated with Rapa in NSC medium without growth factors for 1, 2, 6, 24 and 48 h. At each time point, the expression of mTOR pathway components (total and phosphorylated form of mTOR along with S6K1 and Akt) were analyzed by WB and IFC analysis. Proliferative/metabolic activity of NSC as well as expression of proliferation markers (nestin, CD24 and β1 integrin) were analyzed at a 24-h delay following exposure to Rapa. In a separate set of experiments, differentiation was induced by adding medium containing Shh and retinoic acid after 24 or 48 h of Rapa treatment. Cells were allowed to differentiate for 2 weeks. The expression analysis of differentiation markers (nestin, NF-H, NeuN, MAP2, βIII-tubulin, GFAP, ALDH1L1) was analyzed by WB and IFC analysis. Rapa, rapamycin; Shh, Sonic hedgehog; mTOR, mammalian target of rapamycin; S6K1, ribosomal protein S6 kinase 1; Akt, protein kinase B; NF-M, neurofilament medium chain; NF-H, neurofilament heavy chain; MAP2, microtubule associated protein 2; GFAP, glial fibrillar acid protein; ALDH1L1, aldehyde dehydrogenase 1 family member L1; WB, Western blot; IFC, immunofluorescence.

To elucidate the role of the mTOR pathway in the proliferation of NSCs, iPSC-NPs were seeded in 24 well plates approximately 145,000 cells per well (TPP, Trasadingen, Switzerland) containing coverslips (for immunostaining) or in 6 well plates approximately 300,000 cells per well [for Western Blot (WB)] (TPP, Trasadingen, Switzerland) coated with poly-L-ornithine and laminin with a working density of approximately 60–80% of confluence. After 24 h, the normal NSC medium was changed to NSC medium containing 100 nM rapamycin (Tocris Bioscience, Bristol, UK) and lacking growth factors (“starvation” medium). After 1, 2, 6, 24 and 48 h of treatment, “starvation” medium with Rapa was replaced by normal one and cells were allowed to proliferate for next 24 h. Then, the proliferation activity assay (EdU) or metabolic activity assay (Alamar Blue) was used or cells were washed with PBS and collected for further analysis of proliferation markers by WB and fluorescent immunostaining (IFC). A set of experiments to study proliferative changes was repeated at least 3 times, with corresponding technical replicates in each experiment EdU *n* = 12, Alamar Blue *n* = 4, WB *n* = 3–4, IFC *n* = 2–3. The Rapa treatment algorithm and analysis scheme are shown in Figure 1.

To elucidate the role of the mTOR pathway in the differentiation of NSCs the separate set of experiments was performed. iPSC-NPs were seeded in 24 well plates containing coverslips approximately 145,000 cells per well (for immunostaining) or in 6 well plates approximately 300,000 cells per well (for WB) coated with poly-L-ornithine and laminin with a working density approximately 60–80% of confluence. Next day, the regular NSC medium was changed to “starvation” medium containing 100 nM Rapa and cells were cultured for 24 h and 48 h. After Rapa treatment, differentiation was induced by adding medium consisting of DMEM:F12 and neurobasal medium (1:1), supplements B27 (1:50) and N2 (1:100), a mixture of penicillin and streptomycin antibiotics (1:200) (all from Thermo Fisher Scientific, Waltham, MA, United States), Shh (50 ng/mL), (Pepro Tech, London, United Kingdom), retinoic acid (50 nM) (Sigma-Aldrich, St. Louis, MO, United States), and primocin (1:500) (Invivo Gen, San Diego, CA, United States). The cells were allowed to differentiate for 2 weeks, with half of the medium volume being changed every other day. Then, the cells were washed with PBS and collected for further analysis by WB and IFC. A set of experiments to study proliferative changes was repeated at least 3 times, with corresponding technical replicates in each experiment WB *n*  = 3–4, IFC *n* = 2–3.

### 2.2 Cell proliferation activity (EdU incorporation test)

Proliferative activity of NSC measured by 5-ethynyl-2′-deoxyuridine (EdU) incorporation was assessed using the Click-iT™ Plus EdU Cell Proliferation Kit (Thermo Fisher Scientific, Waltham, MA, United States) according to the manufacturer’s instructions. Briefly, proliferating cells were labeled with 10 μM EdU in NSC medium for 3 h. Cells were then fixed with 3.5% PFA in PBS for 15 min at room temperature (RT), and washed twice with PBS for 5 min. Samples were then permeabilized with 0.5% Triton (Sigma-Aldrich, St. Louis, MO, United States) in PBS for 20 min at RT in the dark. A freshly prepared solution for the detection of EdU was applied for 30 min at RT in the dark. Samples were washed twice with PBS and cell nuclei were visualized by adding DAPI (Sigma-Aldrich, St. Louis, MO, United States) solution prepared in PBS at a dilution of 1:2500. Cell slides were removed from the wells and mounted using Aqua Poly/Mount (Polysciences, Warrington, PA, United States) solution. Stained cells were captured using a Carl Zeiss LSM 880 NLO confocal microscope (Carl Zeiss AG, Oberkochen, Germany). To quantify the staining, 12 images were taken randomly from one sample. The number of EdU- and DAPI-positive nuclei were assessed using Fiji software and presented as a ratio. Values for treated samples were normalized to those for time-matched controls.

### 2.3 Metabolic activity (Alamar blue test)

To evaluate the metabolic activity of NSCs, 24 h after Rapa treatment, culturing in 10% Alamar blue (7-Hydroxy-3H-phenoxazin-3-one-10-oxide sodium salt) (AB) (Sigma-Aldrich, St. Louis, MO, United States) in NSC medium was followed. For this experiment, cells were seeded into 24-well plates at approximately 145,000 cells per well, with 4 technical replicates for each time point. After 3 h of further incubation, the fluorescence level of reduced AB was evaluated using a TECAN GENios microplate reader (Tecan, Männedorf Switzerland) with an excitation wavelength of 550 nm and an emission wavelength of 590 nm (Petrenko et al., 2005). The ratio between the fluorescence of the experimental and blank samples (the same medium without cells) was used as the AB value. Data were presented as relative fluorescence units (RFU). Values for treated samples were normalized to those for time-matched controls.

### 2.4 Fluorescent immunostaining

For immunocytochemical staining, cells were first fixed with 4% paraformaldehyde (Penta, Prague, Czech Republic) and washed with phosphate buffered saline (PBS) (Thermo Fisher Scientific, Waltham, MA, United States). Next, samples were permeabilized with 0.2% Triton (Sigma-Aldrich, St. Louis, MO, United States) in PBS for 10 min at RT in the dark. Non-specific staining was blocked with 10% NGS (Sigma-Aldrich, St. Louis, MO, United States) serum solution in PBS for 30–45 min at RT in the dark. Primary antibodies diluted in 2% NGS and 0.1% Triton in PBS were added and incubated overnight at 4°C in the dark. Individual antibodies and their dilutions are described in Supplementary Table S1. The next day, the corresponding secondary antibodies were diluted 1:400 in the same solution that was used to dilute the primary antibodies, and were used for immunodetection. Cell nuclei were visualized by adding DAPI (Sigma-Aldrich, St. Louis, MO, United States) solution prepared in PBS at a dilution of 1:2,500. Cell slides were removed from the wells and attached to microscopic coverslips using Aqua Poly/Mount (Polysciences, Warrington, PA, United States) solution. Stained cells were captured using a Carl Zeiss LSM 880 NLO confocal microscope (Carl Zeiss AG, Oberkochen, Germany).

### 2.5 Western blotting

The cells were homogenized on ice in radio immunoprecepitation assay (RIPA) lysis buffer (150 mM NaCl, 50 mM Tris (pH 8), 1% Triton X-100, 0.5% sodium deoxycholate, 0.1% sodium dodecyl sulfate) (all from Sigma-Aldrich, St. Louis, MO, United States) containing a protease and phosphatase inhibitor (Thermo Fisher Scientific, Waltham, MA, United States). The homogenized cells were incubated at 4°C for 40 min, then centrifuged at 14,000 RPM and 4°C for 20 min on a 5,804 R centrifuge (Eppendorf, Hamburg, Germany). The resulting samples were frozen at −80°C. The Pierce TM BCA Protein Assay Kit (Thermo Fisher Scientific, Waltham, MA, United States) was used to determine the total protein concentration according to the manufacturer’s instructions. Spectrophotometric measurements were performed using i-control software on an Infinite^®^ 200 PRO Multimode Reader (Tecan, Mannedorf, Switzerland). Samples were immobilized using sodium dodecyl sulfate (SDS) sample buffer [80 mM Tris (pH 6.8), 2% SDS, 10% glycerol, 0.0006% bromophenol blue, 0.1 M DTT] (all from Sigma-Aldrich, St. Louis, MO, United States) for 5 min at 95°C. Electrophoresis was carried out for 20–30 min using Mini-PROTEAN TGX^TM^ Precast Gels (Bio-Rad, Hercules, CA, United States) with a gradient (8–14%) in electrophoresis buffer (25 mM Tris, 192 mM glycine, 0.1% SDS) (all from Sigma-Aldrich, St. Louis, MO, United States). Each well within the gel was loaded with 10 μg of total protein.

Following electrophoresis, proteins were transferred from gels to PVDF membranes (Thermo Fisher Scientific, Waltham, MA, United States) in transfer buffer [25 mM Tris, 192 mM glycine, pH 8.3; along with 20% methanol (v/v) (all from Sigma-Aldrich, St. Louis, MO, United States)] by Western Blot. For the visualization of proteins, membranes were stained with Ponceau S Staining Solution (Cell Signaling Technology, Danvers, MA, United States). Tris-buffered saline / Tween-20 (TBST) buffer (20 mM Tris, 150 nM NaCl, 0.1% Tween 20; pH 7.5) (all from Sigma-Aldrich, St. Louis, MO, United States) was used to wash the membranes between steps.

In order to block nonspecific binding sites, the membranes were incubated with 5% milk solution (Cell Signaling Technology, Danvers, MA, United States). For immunodetection, antibodies were prepared in 5% bovine serum albumin (BSA) solution (Cell Signaling Technology, Danvers, MA, United States) or 5% milk solution (Cell Signaling Technology, Danvers, MA, United States), or directly in TBST and incubated at 4°C overnight. Subsequently, the membranes were washed in TBST and incubated with the appropriate secondary antibodies diluted in TBST for 1 hour on a shaker at RT. The membranes were then washed again with TBST solution. To visualize the staining, Super Signal TM West Dura kit (Thermo Fisher Scientific, Waltham, MA, United States) was used according to the manufacturer’s instructions. Blots were captured on Azure c600 (Azure Biosystems, Dublin, CA, United States) using Capture software. Image digitalization and analysis was performed using Fiji software. The results were normalized for endogenous control proteins: vinculin (124 kDa) or β-actin (45 kDa) (Cell Signaling Technology, Danvers, MA, United States), depending on the molecular weight of the particular protein. At least 3–4 technical replicates were done for each protein at each time point of experiment.

### 2.6 Statistical analysis

All Western Blot data were normalized to the time-matched control—results are presented in pairs: the control sample (taken as 1) and the treated sample normalized to the control. Data was described as the mean SEM for 3 or more independent evaluations. Statistical significance among the various different groups was measured via repeated ANOVA testing.

## 3 Results

### 3.1 Rapamycin treatment affected several upstream and downstream molecules of the mTOR pathway

**Figure 2:**
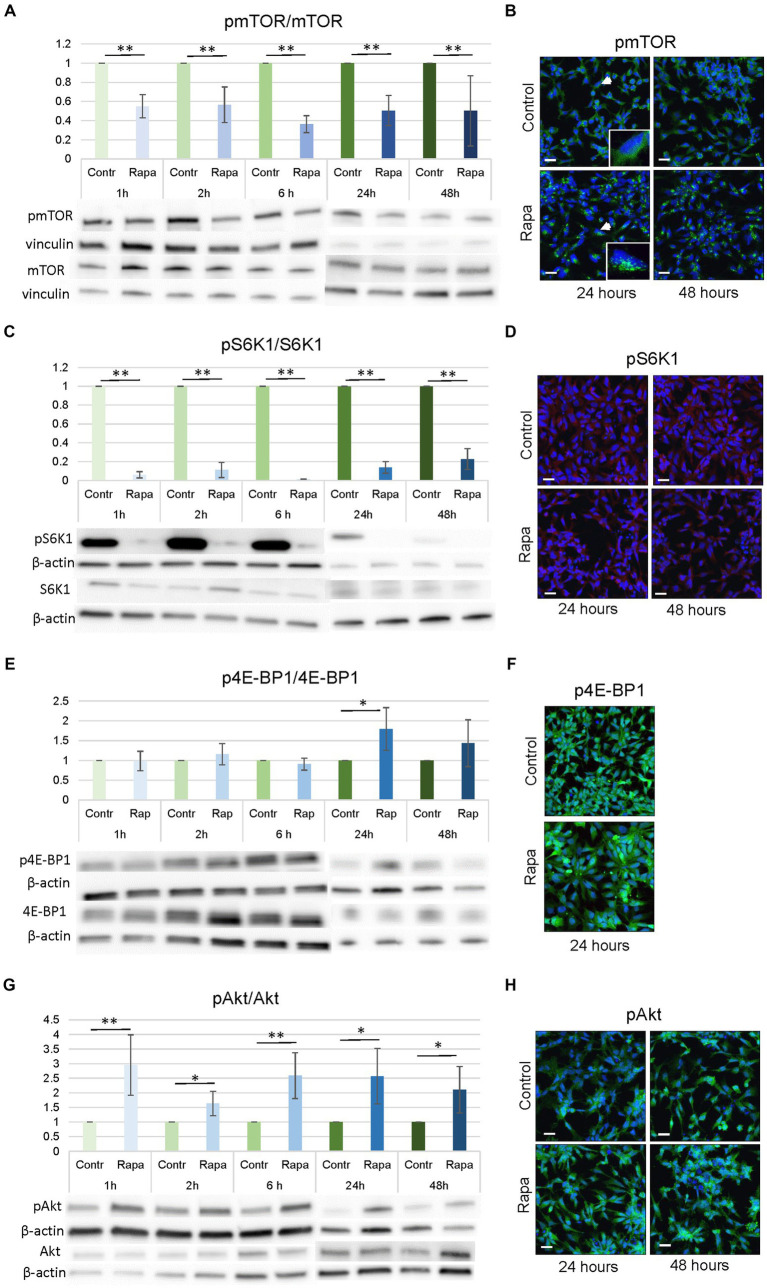
mTORC1 inhibition by rapamycin affected several upstream and downstream molecules of the mTOR pathway. Densitometry analysis revealed that Rapa treatment resulted in a decrease in pmTOR^Ser2448^/mTOR **(A)** and pS6K1^Ser235/236^/S6K1 **(C)** expression ratio and an increase in pAkt ^Ser473^/Akt **(G)** and in pE4BP1/E4BP1 **(E)** expression ratio, however only in one time point – after 24 h of Rapa treatment. WB data were supported by the immunofluorescence staining of NSCs for pmTOR^Ser2448^ **(B)**, pS6K1^Ser235/236^ **(D)**, p4E-BP1 **(F)** and pAkt^Ser473^ **(H)**. All data represent mean ± SEM. The level of statistical significance was marked as follows: ^*^p < 0.05; ^**^p < 0.01. Scale bar = 20 μm. Rapa, rapamycin; mTOR, mammalian target of rapamycin; S6K1, ribosomal protein S6 kinase 1; Akt, protein kinase B; NSC, neural stem cell; WB, Western blot; IFC, immunofluorescence.

mTOR, S6K1, Akt and 4E-BP1 are major components of the mTOR signaling pathway. To elucidate the role of the mTOR pathway in the process of neural stem cell differentiation, neural stem cells (NSC) were treated with rapamycin (100 nM) for 1, 2, 6, 24, and 48 h. Results showed that Rapa significantly reduced the relative ratio of pmTOR/mTOR by approximately 50% compared to the corresponding time-matched controls at all time points (*p < 0.05, **p < 0.01; Figure 2A).

As shown in Figure 2, WB analysis results are presented as controls and corresponding Rapa-treated groups, with the control values set to 1. Immunofluorescence (IFC) of pmTOR showed that Rapa treatment resulted in a shift in cellular localization. In untreated NSC, pmTOR was evenly distributed in the cell cytoplasm, however, after treatment with Rapa for 24 or 48 h, the localization of pmTOR changed as it concentrated close to the cell nuclei (Figure 2B). Similar patterns were observed after a shorter time treatment period (data not shown). These data indicate effective inhibition of mTORC1 by the above concentration of Rapa at all experimental time points.

Rapamycin is a potent inhibitor of mTORC1, therefore we studied the status of downstream targets of mTORC1, S6K1, and 4E-BP1, after Rapa treatments. Protein expression level analysis (WB) showed that Rapa treatment significantly inhibited pS6K1^Ser235/236^/S6K1 levels compared to the time-matched controls at all time points (−90%; **p < 0.01) (Figure 2C). IFC staining analysis remained inconclusive for pS6K1^Ser235/236^ (Figure 2D). Western blotting analysis showed that the expression of p4E-BP1/4E-BP1 generally remained unaffected after Rapa treatments, only 24 h after the rapamycin treatment, a significant increase in the ratio of p4E-BP1/4E-BP1 compared to the corresponding controls (*p* = 0.0255) was seen (Figure 2E). IFC staining for p4E-BP1 is shown on Figure 2F. The phosphorylation of Akt on S473, which is regulated by mTORC2, and the results showed that the Rapa treatment caused an increase in pAkt/Akt ratio as a result of the mTORC1 inhibition (Figure 2G). The intensity of the increase in pAkt/Akt expression was approximately 15–30% higher and was statistically significant (*p < 0.05, **p < 0.01) at all time points after Rapa treatment compared to controls (Figure 2G). IFC staining also confirmed the noticeably higher expression of pAkt^Ser473^ at 24 and 48 h after Rapa treatment (Figure 2H).

These results implied that mTORC1 inhibition by Rapa inhibited the activated mTOR as well as downstream molecules of the mTOR pathway, S6K1 and, to a lesser extent, 4E-BP1. In addition, up-regulation of Akt^Ser473^ phosphorylation following mTORC1 inhibition resulted in an increased pAkt/Akt ratio (Figure 2G). In summary, our data indicate that numerous molecules within the mTOR pathway, both downstream and upstream, were significantly inhibited following treatment with Rapa. This signifies the successful establishment of an mTOR inhibition model in NSC culture, enabling us to investigate the role of the mTOR pathway in NSC proliferation and differentiation.

### 3.2 Rapamycin suppressed NSC proliferation and metabolic activity

**Figure 3:**
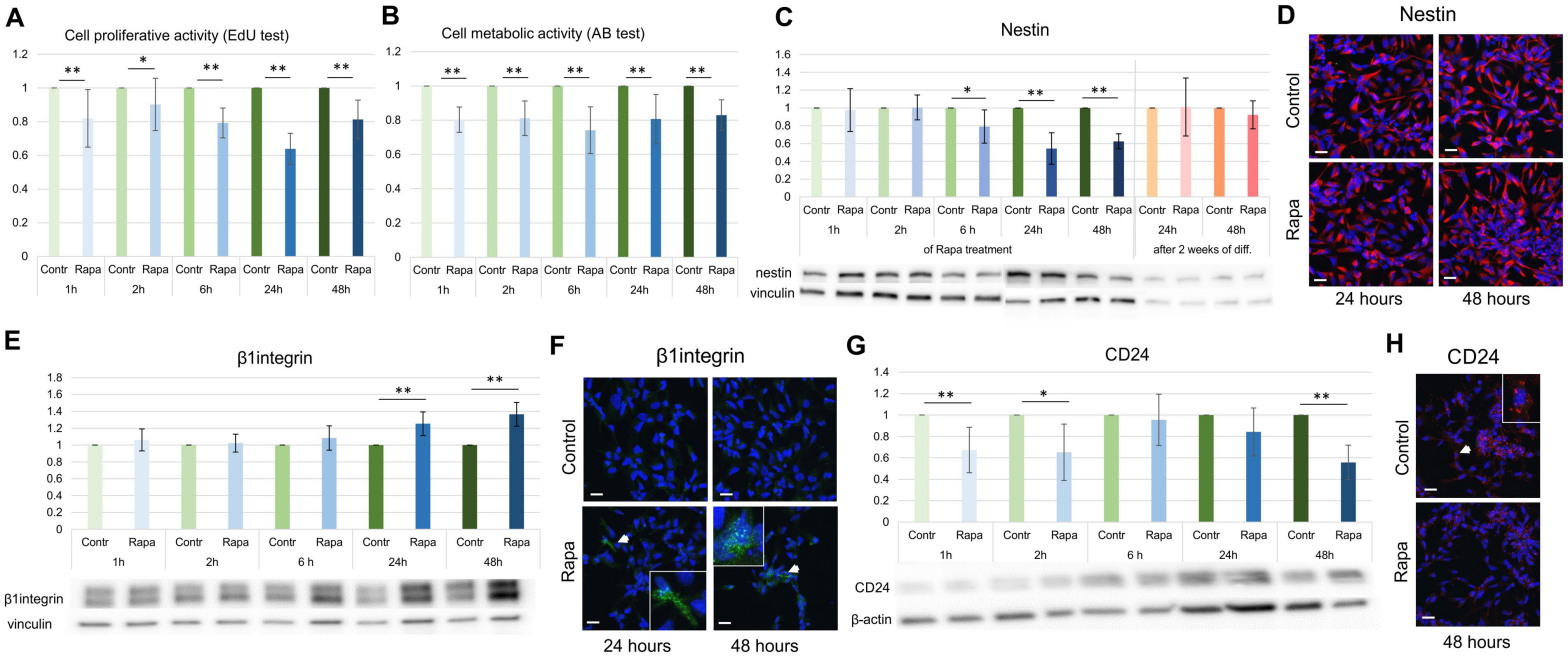
mTORC1 inhibition suppressed NSC proliferation. Proliferative activity of NSCs, as assessed by EdU-incorporation assay at a 24 h delay after Rapa treatment was significantly reduced compared to controls **(A)**. The Alamar blue assay also demonstrated a significant reduction in NSC metabolic activity following mTORC1 inhibition **(B)**. WB and IFC analysis depicting a significant decrease in nestin expression at 6, 24 and 48 h **(C,D)**; an increase in β1integrin expression was observed at 24 and 48 h after Rapa treatment **(E,F)**. Rapa treatment suppressed CD24 expression at 1, 2 and 48 h **(G,H)**. All data represent mean ± SEM. The level of statistical significance was marked as follows: ^*^p < 0.05; ^**^p < 0.01. Scale bar = 20 μm. Rapa, rapamycin; NSCs, neural stem cells; WB, Western blot; IFC, immunofluorescence.

The gold standard in the estimation of cell proliferation is bromodeoxyuridine (BrdU), which is able to incorporate into the replicating DNA of cells during their S-phase. In this study we used EdU as an analog of BrdU analysis. Among other markers, we selected CD24, nestin and β1integrin, which are considered markers of proliferation due to their involvement in cell division (Calaora et al., 1996; Blaess et al., 2004; Park et al., 2010; Myers et al., 2011; Yan and Cui, 2023). To demonstrate the effect of mTORC1 inhibition on NSC proliferation, the EdU assay was used after Rapa treatment for different time periods: 1, 2, 6, 24 and 48 h. The proliferation test done immediately after the application of Rapa showed no significant difference between the activity of the treated versus control cell populations, irrespective of the times after treatment. However, a delayed assay (performed 24 h after Rapa treatment) showed a significant decrease in the proliferative activity of NSCs starting from 1 h of mTOR inhibition (Figure 3A).

Using a similar time schedule, we analyzed the metabolic activity of NSCs after Rapa treatment. The Alamar blue test results showed a significant reduction in metabolic activity corroborating results obtained with EdU proliferation assay (Figure 3B).

Evaluation of the expression of several proliferation markers revealed their significant changes at different time points of the study. Expression levels of nestin, a marker of undifferentiated neural stem cells or progenitor cells (Yiannis et al., 2021), was reduced compared to the time-matched controls after 6, 24 and 48 h of Rapa treatment. However, the nestin level returned to normal values 2 weeks after the withdrawal of Rapa treatment for 24 or 48 h, in the differentiation condition medium (Figure 3C).

The expression of β1integrin, a vital member of the integrin family crucial for NSC proliferation, migration, differentiation, axon guidance, and growth (Georges-Labouesse et al., 1998; Blaess et al., 2004; Leone et al., 2005; Myers et al., 2011; Yan and Cui, 2023) was significantly upregulated after 24 and 48 h of mTORC1 inhibition (Figure 3E). Contrastingly, the levels of CD24, whose expression is linked to the regulation of neural stem/progenitor cell proliferation (Calaora et al., 1996; Poncet et al., 1996), were notably diminished following 1, 2, and 48 h of Rapa treatment (Figure 3G) when compared to time-matched controls. As shown in Figure 3, all WB analysis results, as well as the metabolic activity of the cells, are presented in pairs: a control sample and a treated sample, normalized to the corresponding time control.

IFC analysis supported the WB findings (decrease in nestin, and CD24, and increase β1 integrin) of control versus Rapa treated NSC culture (Figures 3D,F,H). These results suggest that inhibition of mTORC1 suppressed the NSC proliferation, based on both the proliferative and metabolic activity and the expression of proliferative markers.

### 3.3 mTOR inhibition affected neural stem cell differentiation towards neuronal linage

In addition to βIII-tubulin, which is normally expressed by immature neurons, MAP2, NeuN, NF-H, and NF-M are considered as the markers of mature neurons. MAP2 serves multiple functions, including nucleating and stabilizing microtubules, regulating organelle transport within axons and dendrites, and anchoring regulatory proteins such as protein kinases, which are vital for signal transduction. NF-H, and NF-M are intermediate filaments primarily responsible for providing structural support in neuronal axons. βIII-tubulin plays a critical role, particularly in neuronal differentiation, migration, neurite outgrowth, and axon formation. While all the markers mentioned above belong to structural proteins located in the cell cytoplasm, NeuN stands out as a nuclear protein widely employed as a marker for mature neurons. It is involved in regulating gene expression and promoting neuronal maturation.

**Figure 4:**
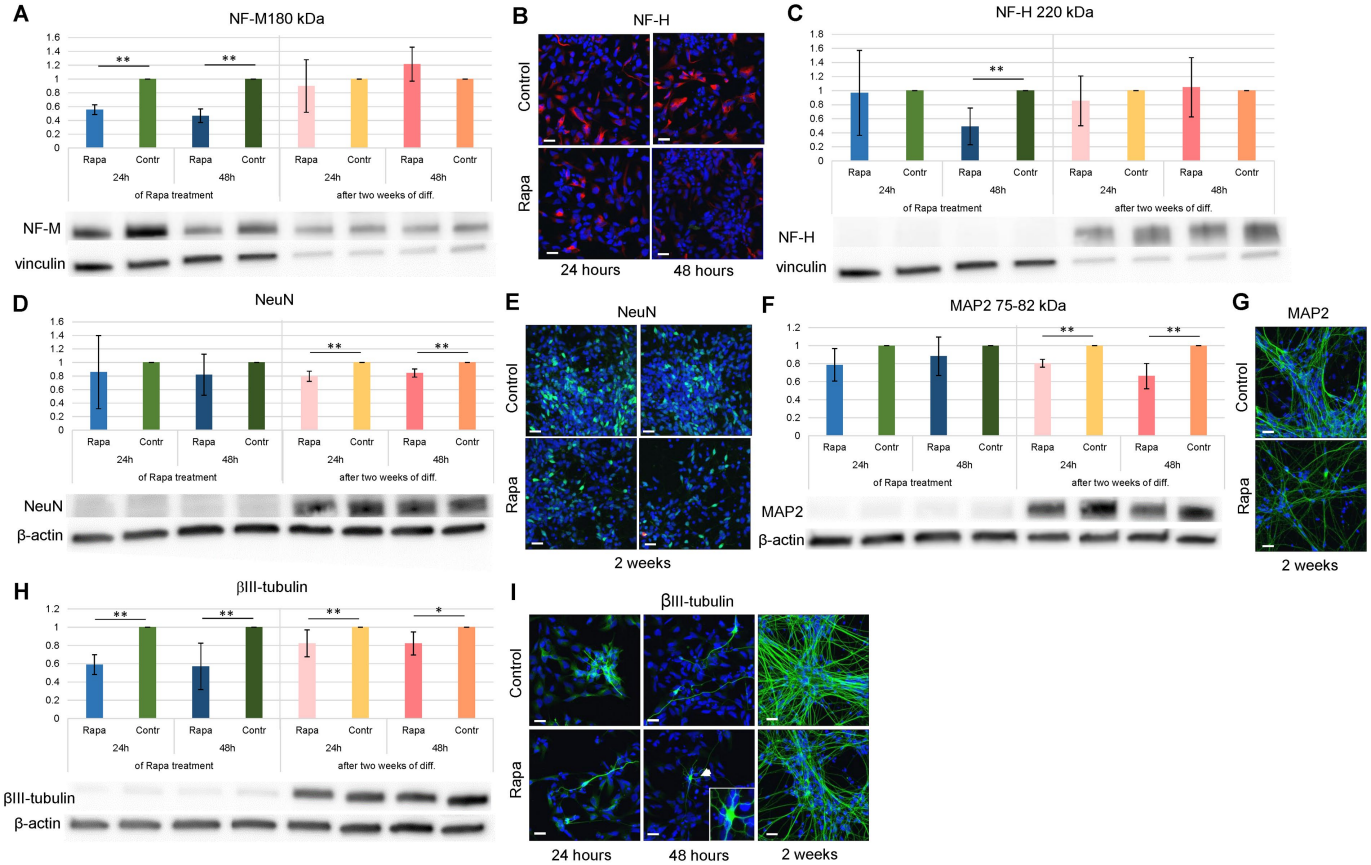
Neuronal differentiation of rapamycin treated NSCs. Rapa treatment caused alterations in the expression of neuronal markers within 2 weeks of NSC differentiation. The expression of NF-M 180 kDa **(A)**, NF-H 220 kDa **(C)** and βIII-tubulin **(H)** was significantly decreased as compared to the corresponding time-matched controls immediately following 24–48 h of Rapa treatment, while the expression of NeuN **(D)** and MAP2 **(F)** at these time points remained unaltered. Within 2 weeks of neuronal differentiation (presented as dark and light pink bars), Rapa treated cells displayed a significant reduction in expression of NeuN **(D)**, MAP2 **(F)** as well as βIII-tubulin **(H)**, while expression of NF-H and NF-M remained unchanged. WB analysis was supported by IFC staining for markers of NSC differentiation: NF-H **(B)**, NeuN **(E)**, MAP2 **(G)** and βIII-tubulin **(I)**. All data represent mean ± SEM. The level of statistical significance was marked as follows: ^*^p < 0.05; ^**^p < 0.01. Scale bar = 20 μm. Rapa, rapamycin; NF-M, neurofilament medium chain; NF-H, neurofilament heavy chain; MAP2, microtubule associated protein 2; WB, Western blot; IFC, immunofluorescence.

To elucidate the role of the mTOR signaling pathway in neuron formation, we studied the expression of the aforementioned proteins after Rapa treatment and further differentiation of NSCs. Investigation of the inhibition of mTORC1 leading to the suppression of neuronal differentiation of NSC showed that as early as 24 h after Rapa application, the expression of NF-M 180 kDa (Figure 4A) and βIII-tubulin (Figure 4H) was significantly lower compared to the corresponding time-matched controls.

A similar effect was observed after 48-h treatment with Rapa, when the level of NF-H 220 kDa was also reduced (Figure 4C). Both treatment times, 24 and 48 h, also resulted in a long-term effect on neural stem cell differentiation. Two weeks after the initiation of differentiation, the expression of neuronal specific markers NeuN (Figure 4D), MAP2 (Figure 4F) and βIII-tubulin (Figure 4H) was significantly reduced compared to the respective controls, and regardless of the initial duration of Rapa treatment. WB analysis was supported by IFC staining (Figures 4B,E,G,I). A significant reduction in the expression of several neurospecific proteins suggests that mTOR inhibition suppresses the differentiation of neural stem cells towards neurons.

### 3.4 mTOR inhibition influenced neural stem cell differentiation toward glial linage

**Figure 5:**
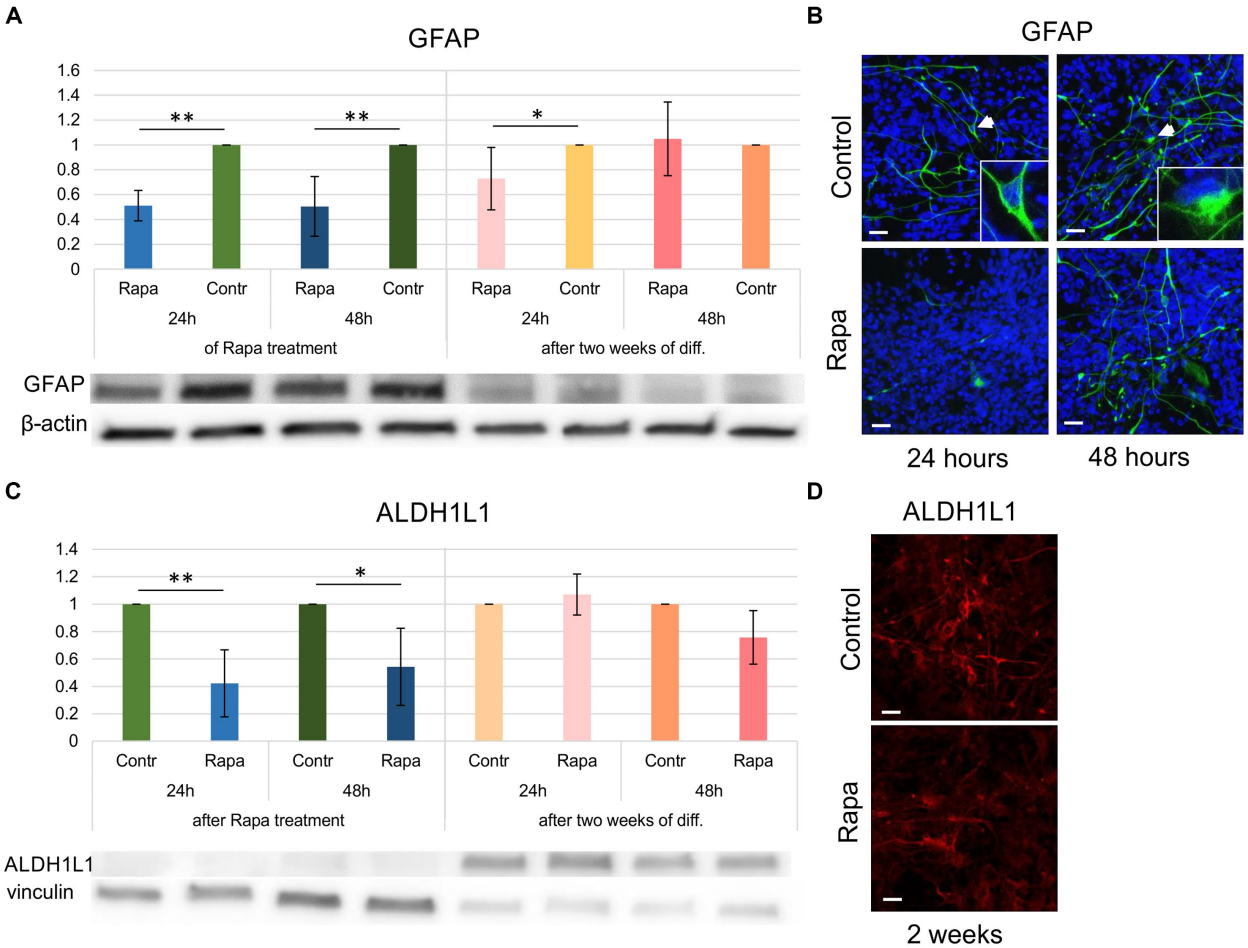
Glial differentiation of rapamycin-treated NSCs. The expression GFAP **(A)** as well as ALDH1L1 **(C)** immediately following 24–48 h of Rapa treatment was significantly reduced compared to the time-matched controls. Within 2 weeks of glial differentiation (presented as dark and light pink bars), Rapa-treated cells displayed a significant reduction in GFAP **(A)**. WB analysis was supported by IFC staining of 2 weeks differentiated cells **(B,D)**. All data represent mean ± SEM. The level of statistical significance was marked as follows: ^*^p < 0.05; ^**^p < 0.01. Scale bar = 20 μm. Rapa, rapamycin; GFAP, glial fibrillar acid protein; ALDH1L1, aldehyde dehydrogenase 1 family member L1; WB, Western blot; IFC, immunofluorescence.

Differentiation of glial lineages was also impaired as a result of mTOR inhibition. GFAP and ALDH1L1 (aldehyde dehydrogenase 1 family member L1) expression was affected by Rapa treatment—with a significant reduction immediately after 24–48 h of treatment compared to time-matched controls (Figures 5A,C). Within 2 weeks of differentiation, the number of GFAP-positive cells (in both control and Rapa-treated cultures) was relatively low compared to the undifferentiated culture. The question remains whether the decrease in GFAP is due to differentiation of neural precursors into neurons or to the effect of Rapa. However, the fact that despite the low level of specific proteins, it was possible to determine that the GFAP level in the Rapa-treated NSC culture for 24 h was significantly lower compared to the time-matched control suggests that some effect on the cytoskeleton may have occurred after Rapa treatment. Longer Rapa treatment (48 h) had no effect on GFAP protein levels during the 2 weeks of differentiation. ALDH1L1 expression was not affected by Rapa treatment after 2 weeks of differentiation. WB results were supported by immunofluorescent staining (Figures 5B,D). Our findings reveal a subtle impact of mTOR pathway inhibition on the expression of glial markers. Given the overall low proportion of glial cells within the 2-week differentiated cell culture, we propose that glial differentiation may also be impaired by Rapa. However, further investigation is necessary to fully elucidate this potential effect.

## 4 Discussion

mTOR plays a crucial role in the development of the central nervous system, as it is involved in various physiological processes such as learning, memory formation, synaptic plasticity, development, and the differentiation of neurons (Gangloff et al., 2004; Hartman et al., 2013; Garza-Lombó and Gonsebatt, 2016). In parallel, neurological disorders associated with dysfunction of the mTOR pathway have been reported in epilepsy, autism, and neurodegenerative diseases. Several studies have attempted to describe the involvement of mTOR complexes in the proliferation and differentiation of NSCs, with conflicting findings; however, its mechanism of action remains to be elucidated. Our results demonstrated the effect of mTORC1 inhibition on the proliferation and differentiation of NSCs *in vitro*. We focused on the short-term and long-term effects of mTORC1 suppression on neural differentiation and partly elucidated the mechanism of these processes. mTOR inhibitors are reported to be effective suppressors of malignant cell proliferation and are widely used even in clinical practice. Moreover, Kassai et al. (2014) demonstrated that mTORC1 signaling exhibits distinct stage-specific functions in developing and adult neurons, indicating the involvement of different downstream effectors controlling cellular processes in embryonic and adult neurons. Despite the observations and conclusions of several investigators, demonstrating the role of mTORC1 signaling is to direct NSC differentiation rather than to induce proliferation (Crowell et al., 2015; Mahoney et al., 2016), our results clearly demonstrated that mTORC1 inhibition affects both physiological processes with respect to distinct time frames.

Our results demonstrated that Rapa treatment led to a significant downregulation of phosphorylation of pmTOR^Ser2448^ and pS6K1^Ser235/236^, and revealed an enhanced phosphorylation of Akt on S473. Studies have demonstrated that mTOR may function via nucleocytoplasmic signaling, where maximal phosphorylation of S6K1 is required (Bachmann et al., 2006; Ahmed et al., 2019). Our results show that the cellular localization of mTOR is altered following Rapa treatment (Figure 2A). In control NSCs, the localization of mTOR was in the cytoplasm, which changed after treatment with Rap. mTOR translocation to the nucleus upon Rapa treatment induction appeared to be rapid since it is documented within a few minutes of treatment (Jhanwar-Uniyal et al., 2013). The exact role of mTOR in the nucleus remains to be investigated; however, activated mTOR has been shown to be localized to subnuclear structures that resemble polymorphonuclear (PML) bodies. These PML bodies represent distinct, dynamic structures that control cell proliferation, apoptosis, and cellular senescence that are also linked with the Akt phosphorylation (Salomoni and Pandolfi, 2002).

The results demonstrate that p4E-BP1 level was significantly upregulated 24 h of Rapa treatment. It was reported that while short-term inhibition with rapamycin resulted in dephosphorylation of 4E-BP1 and its association with eIF4E, long-term exposure for 24 and 48 h resulted in hyperphosphorylation and dissociation of eIF4E. This phosphorylation occurred despite ongoing inhibition of mTORC1, as demonstrated by the results for S6 kinases. These were inhibited continuously 24 h after the addition of rapamycin (Choo et al., 2008). These conclusions agree with our observed results, when we described persistent S6 inhibition and hyperphosphorylation of 4E-BP1. Aberrant mTOR signaling is associated with numerous disorders and the mTORC1 is essential in many cellular processes, including protein synthesis, cell viability, metabolism, survival, catabolism, autophagy and others. These functions are carried out by two crucial molecules, S6K and 4E-BP1 which are phosphorylated by mTORC1 to regulate translation and protein synthesis (Burnett et al., 1998). Multiple studies have demonstrated that the suppression of the mTOR/S6K signaling pathway leads to the inhibition of cell proliferation and as well as induction of apoptosis and autophagy. It is important to note that Rapa differentially inhibits S6K and 4E-BP1 to mediate cell type-specific repression of mRNA translation (Choo et al., 2008). The rapalog-FKBP12 complex reduces mTORC1 kinase activity in a variable manner on its different substrates, with S6K among the most sensitive, and 4E-BP1 among the most resistant, to rapalog inhibition.

The Rapa-insensitive, Rictor-containing complex (mTORC2) is implicated in actin cytoskeleton regulation, as well as the phosphorylation of Akt on Ser473. Various studies have revealed the existence of several negative feedback loops originating from p70S6K1 to Akt, which is associated with Rapa or rapalog treatments (Julien et al., 2010; Jhanwar-Uniyal et al., 2013). In fact, inhibition of p-p70S6K1 by rapalog activates insulin receptor substrate 1 (IRS-1) and phosphatidylinositol 3 kinase (PI3K), by lifting negative feedback, resulting in the phosphorylated activation of Akt at Thr308 (Carracedo et al., 2008). Inhibition of p-p70S6K1 can lead to phosphorylation of Rictor at Thr1135, resulting in the dissociation of Rictor from mTORC2 to promote the phosphorylation of Akt at Ser473, the direct downstream targets of Rictor, indicating that mTORC1 negatively regulates mTORC2 via the phosphorylation of Rictor at Thr1135 (Breuleux et al., 2009). Biochemical and genetic evidence has demonstrated that mTORC2 phosphorylates Akt at Ser473. These observations suggest that the inhibition of mTORC1 by Rapa or rapalogs can reactivate Akt signaling by two distinct mechanisms, Akt^Thr308^ occurs by activation of the p70S6K1/IRS-1/PI3K and Akt^Ser473^ by triggering p70S6K1/mTORC2 feedback loops. It appears that the negative regulation of mTORC2 by mTORC1 can occur within a short period of time, as 30 min pre-treatment with Rapa-induced insulin-dependent phosphorylation of Akt^Ser473^ (Gulati et al., 2009). This implies that the mTORC1-induced inhibitory response leading to negative regulation of mTORC2 activity occurs in a rapid manner. This observation is consistent with our results, that enhanced phosphorylation of Akt^Ser473^ occurs within 1 h of Rapa treatment (Figure 2C).

The results demonstrated that the proliferative activity of NSCs was suppressed as early as 1-h post-Rapa treatment (Figure 3A). Long-term treatment sustained a significant decrease in the metabolic activity of NSCs, with the strongest effect seen after 24 h of long-term Rapa treatment when a decrease in cell proliferative activity of approximately 40% was reported, compared to untreated cells. 48 h of Rapa treatment appears to be less effective compared to other times. Similarly, the metabolic activity of NSCs was also suppressed; however, its minimum value was observed as a result of 6-h Rapa treatment (Figure 3B). It is possible that as the metabolic activity of cells depends not only on the mTOR signaling pathway, but the effect of mTOR inhibition could also be compensated by alternate metabolic pathways during this time period. These results corroborate the finding that 24- and 48-h treatment with Rapa had a greater effect on the expression of some proteins associated with the proliferation of NSCs compared to a shorter treatment period (Figures 3B–G).

Nestin, a type VI intermediate filament protein, is expressed by many cell types during development, although its expression is usually transient and absent in the adult brain. It is a marker of neural stem cells or progenitor cells; its expression dissipates after differentiation (Yiannis et al., 2021). During neuro- and gliogenesis, nestin is replaced by cell-type-specific intermediate filaments, i.e., neurofilaments and glial fibrillar acidic protein (GFAP). Nestin expression can be re-induced in adults under pathological conditions such as glial scar formation after injury to the central nervous system and during the regeneration of injured nervous tissue. The physiological function of nestin *in vivo* remains elusive. Since its discovery, nestin has been unequivocally accepted as an NSC marker both during embryonic development as well as in the adult brain (Lendahl et al., 1990; Gilyarov, 2008). Numerous *in vivo* and *in vitro* studies currently rely on nestin expression to track proliferation, migration, and the survival of stem cells, and its expression dissipates post-differentiation (Park et al., 2010). Our results demonstrated that following at least 6 h of Rapa treatment nestin expression decreased, and after 24 h of Rapa treatment, nestin expression was reduced to 50% compared to the control. However, in 2 weeks of differentiation after Rapa treatment, the expression of nestin returned to levels measured in the control cells (Figure 3B). These initial reduction in nestin levels suggest that stem cell differentiation has taken place.

CD24 is a highly glycosylated protein with a small protein core that is associated with the plasma membrane via a glycosyl-phosphatidylinositol anchor which expressed on the surface of most differentiating neuroblasts. It interacts with a wide range of downstream signaling networks such as mitogen-activated kinase signaling, nuclear factor-κB signaling, Notch and Hedgehog signaling to mediate various functions such as nerve migration, neurite outgrowth and neurogenesis. CD24 is dynamically expressed during the development of the rodent (Calaora et al., 1996) and human (Poncet et al., 1996) nervous system. Consistent with its expression in areas of secondary neurogenesis, CD24 is also associated with the regulation of neural stem/progenitor cell proliferation. Our results show that even a 1-h treatment of NSCs with Rapa caused a decrease in CD24 expression. Similar effects were observed 2 and 48 h after Rapa application.

Integrin beta-1 (β1 integrin), also known as CD29, is a cell surface receptor. It belongs to the group of transmembrane heterodimeric proteins that govern most of the interactions between cells and the extracellular matrix (Hynes, 2002). The β1 integrins, which constitute the largest subgroup of integrins play a significant role in the development and regeneration of the CNS. They are important for NSC proliferation, migration, differentiation, axon guidance, and growth (Georges-Labouesse et al., 1998; Blaess et al., 2004; Leone et al., 2005; Myers et al., 2011; Yan and Cui, 2023). Our results demonstrated that mTOR inhibition resulted in an increase in β1 integrin expression. The strongest effect was observed 48 h after treatment with Rapa. Although the relationship between β1 integrin and the components of the mTOR signaling pathway in NSC regulation has not been described, several studies suggest that β1 integrin expression and pAkt/Akt activity are in direct correlation, in cancer models (Virtakoivu et al., 2012; Bui et al., 2019). These observations support our findings indicating that pAkt and β1 integrin levels are both elevated following 24/48 h of mTORC1 inhibition. However, in addition to proliferation, β1 integrin plays a crucial role in the formation of focal adhesions, which are essential for proper cell division and migration. Since these processes are activated during initial differentiation, which occurs within the first 24/48 h in culture, the observed upregulation in β1 integrin expression may be attributed to changes in adhesion and migration rather than proliferation. Recent studies have highlighted the relevance of considering β1integrin isoforms. Studies indicate that the β1 integrin-1B isoform acts as a dominant negative, and inhibits cell adhesion (Balzac et al., 1994). Another isoform, beta-1C, has been shown to have an inhibitory effect on DNA synthesis in the G1 phase of the cell cycle (Meredith et al., 1995). This may contribute to a slowdown of the growth phase and the initiation of differentiation processes in NSC culture. Further studies are necessary to elucidate the involvement of β1 integrin in proliferation and differentiation of NSC. Taken together, our data suggest that inhibition of mTORC1 activity significantly suppressed NSC proliferation and metabolic activity *in vitro*. As mentioned above, mTORC2 is known to be one of the triggers for cytoskeletal remodeling (Saxton and Sabatini, 2017). Our results are consistent with this observation and demonstrated a significant decrease in the expression of proteins belonging to the NSC cytoskeletal proteins, such as βIII-tubulin, NF-H, and GFAP after 24–48 h of mTORC1 inhibition.

βIII-tubulin, a microtubule element exclusively expressed in neurons and neural cells, is actively involved in the regulation of ligand interactions and the formation of microtubules. Its deficiency reduces microtubule dynamics in growth cones, which reduces axon growth after peripheral nerve injury and strongly delays functional recovery (Latremoliere et al., 2018). As demonstrated by Ferreira and Caceres (1992), βIII-tubulin isotypes are not a major factor involved in the regulation of microtubule assembly during early neurite outgrowth, but play an important role in maintaining further neurite elongation and/or defining a unique binding property of MAP to specific subsets of microtubules. Our results show that mTOR inhibition resulted in a significant decrease in βIII-tubulin expression compared to time-matched controls at all observation time points, suggesting that mTORC1 suppression targets the earliest marker of neuronal differentiation.

Neurofilaments (NF) are intermediate filaments found in the cytoplasm of neurons with a higher density in axons, and their number correlates with the diameter and maturation of axons and their myelination. At the beginning of development, the axons are narrow processes containing relatively few neurofilaments (Lendahl et al., 1990). Those axons that become myelinated accumulate more neurofilaments, which leads to an increase in their caliber. The diameter of an axon can increase fivefold after it grows and fuses with a target cell. The light chain (NF-L), medium chain (NF-M), and heavy chain (NF-H) of neurofilaments appear gradually during neuronal development. They replace nestin with transiently expressed vimentin (Pachter and Liem, 1984). The tail domains of the high molecular weight NF proteins (NF-M and NF-H) are composed of lysine-serine-proline (KSP) repeats, which have been shown to be highly phosphorylated in the axon and play a role in mediating neuron-specific properties, including axon gauge and conduction velocity. Phosphorylation of KSP motifs in peptide substrates is regulated by mitogen-activated protein kinases (extracellular signal-regulated kinases, Erk1/Erk2) *in vitro* and *in vivo* (Li et al., 1999), which are in crosstalk with the mTOR signaling pathway. Our results showed that the expression of NF-M and NF-H was significantly reduced compared to the controls after 24- and 48-h Rapa treatment, implying that mTORC1 inhibition caused a disturbance in axonal maturation.

GFAP, a type III intermediate filament protein, is expressed primarily by astrocytes and plays a significant role in the interactions between astrocytes and neurons. Recent studies have demonstrated that GFAP is also very important for cell migration, proliferation, vesicle transport/autophagy, synaptic plasticity, and neurite outgrowth (Triolo et al., 2006; Yoshida et al., 2007; Potokar et al., 2010). These observations originated from research that focused on mature astrocytes, where GFAP is the main marker and structural component of the cytoskeleton. Our results showed that mTOR inhibition resulted in reduction in GFAP expression, which persisted even after an initial time period of differentiation. It should be noted that in our experiments, which used NSC culture rather than mature neurons and astrocytes, we suggest that the dysregulation of GFAP expression may primarily cause dysregulation of neurite proliferation, migration, and growth.

All three of these proteins (NF-H, βIII-tubulin and GFAP) were significantly reduced within 24–48 h as a result of the Rapa treatment. Considering all of the above, we conclude that long-term inhibition of mTORC1 leads to the inhibition of mTORC2. We explain the mechanism of mTORC1 and mTORC2 cooperation in our study as follows. mTORC2 shares some common subunits with mTORC1, including mTOR, Deptor and mLST8. Further studies have revealed that mLST8 is essential for maintaining the Rictor-mTOR interaction also in mTORC2 complex, suggesting that mLST8 might be an important molecule for both mTOR complexes and may regulate the dynamic balance of these complexes in mammalian cells. Inhibition of mTORC1 by rapamycin has different effect, depending on the cell type, as it can enhance mTORC2 activation (Gulati et al., 2009) or suppress mTORC2 activation (Sarbassov et al., 2005). These differences in response are attributed to the substrate assembly and de assembly. In correlation with the decrease in the expression of several neurospecific proteins, which are also part of the cell cytoskeleton, we hypothesize that this could lead to the destabilization of the NSC cytoskeleton and, consequently, to the inability to further differentiate into neurons.

Our results display that after 2 weeks of NSC differentiation, the level of expression of several major neuron-specific proteins (NeuN, NF-M, NF-H, βIII-tubulin, MAP2) was 6–800 times higher than the level before differentiation. At the same time, the level of glial markers ALDH1L1 and S100β (data not shown) were increased only 3–4 times, whereas level of GFAP was decreased by about 10–15 times compared to the undifferentiated cells. These results suggest that the differentiation protocol employed yielded a culture with a higher proportion of neurons than astrocytes. Due to the notably greater impairment in the expression of neuronal-specific markers NeuN, MAP2c, and βIII-tubulin compared to glial markers following 2 weeks of differentiation post-Rapa treatment, we assumed that mTOR inhibition primarily suppresses neuronal lineage differentiation.

NeuN, a neuronal nuclear antigen is mostly expressed in mature neurons. Figure 4 demonstrates that the expression of NeuN increased only 6-fold compared to the undifferentiated cells, while the expression of other neuron-specific markers (NF-H) increased about 600-800-fold, suggesting that culture of neuronal cells may represent different stages of neuronal development, i.e., not exclusively mature neurons. Nevertheless, NeuN levels were significantly altered by previous mTOR inhibition.

MAP2, which is expressed both during development and in adulthood, is known to play a pivotal role in microtubule assembly, a vital step in neurogenesis. The MAP2c (about 70 kDa) that we investigated in this study, is a juvenile isoform that is downregulated after early neuronal development (Garner et al., 1988), while MAP2b is expressed both during development and in adulthood. MAP2a starts to be expressed when MAP2c levels fall and is not found uniformly in all mature neurons (Chung et al., 2002). The fact that we observed in our experiment that the level of MAP2c remains relatively high 2 weeks after the onset of NSC differentiation is further confirmation that a higher proportion of immature NSCs is evident at this time.

Aldehyde dehydrogenase family 1, member L1 (ALDH1L1) serves as a pan-astrocytic marker, exhibiting a homogeneous expression throughout the brain and other part of nervous system. This protein plays a crucial role in various biochemical reactions, including *de novo* nucleotide biosynthesis and methionine regeneration, making it influential in cell division and growth processes. The utilization of ALDH1L1 as an astrocyte marker was initially proposed based on data indicating its high, broad, and specific expression across nearly all astrocytes, distinguishing them from other CNS cell types (Cahoy et al., 2008; Doyle et al., 2008; Dougherty et al., 2010). Our results revealed a significant dysregulation in the expression of ALDH1L1 immediately following prolonged (24 or 48 h) inhibition of mTOR. However, this effect diminished over the course of 2 weeks of differentiation.

All in all, our results suggest that gliogenesis was also affected by the long-term influence of mTOR inhibition since GFAP expression was reduced following 2 weeks of differentiation. Although the proportion of glial cells in the differentiated culture was noticeably low, further studies should confirm this assumption.

Neurogenesis requires an orchestrated interaction of inductive signals as well as secreted signals, leading to the formation of NSCs. The PI3K/Akt/mTOR pathway plays a crucial role in the proper development of cortical layers and differentiation. Hyperactivation of this axis leads to several developmental disorders. In addition, mutations of mTOR can cause cortical delamination and dysmorphic neurons (Mirzaa et al., 2016). Furthermore, the mTOR complexes mTORC1 and mTORC2, play a key role in stem cell maintenance, albeit a diverse one, primarily due to their different downstream targets. mTORC1/p70S6K1 is maintained at lower levels than mTORC2 in embryonic stem cells (ESCs), through TSC1/2 activation. In ESCs, lower levels of p70S6K1 have been shown to be associated with the maintenance of their undifferentiated state, primarily due to the reduced translation rate. Furthermore, loss of stemness is achieved by elevated mTORC1 signaling due to the knockdown of TSC2, which causes an increase in protein synthesis (Easley et al., 2010). Also, dual inhibitors of both complexes of mTOR halted the stem cell self-renewal of the GBM stem cell (Jhanwar-Uniyal et al., 2022). The findings of our study strengthen the role of mTOR pathway in the regulation of NSC.

This study has some limitations. Quantitative analysis of protein marker expression was performed only by WB and these findings were not confirmed by ICC quantification for various reasons. In particular, the different sensitivities of the antibodies used for the two types of analysis led to incongruent results. WB was preferred due to its generally higher sensitivity and objectivity in quantification compared to ICC staining. WB is able to distinguish between different molecular forms of proteins and capture dynamic changes in their expression over time, as has been shown for markers such as MAP2 and NF-M or NF-H. In contrast, ICC with the same antibody tends to stain all together without this level of resolution. The differences between ICC and WB quantification can also be related to the differentiation process. During the two-week period of differentiation, the population of cells in culture proliferates, leading to overlapping cellular elements, especially those with fibrillar structures. As a consequence, ICC quantification in such cultures becomes less objective and more prone to bias.

## 5 Conclusion

Despite a significant number of studies elucidating the role of the mTOR signaling pathway in the proliferation and differentiation of NSCs, the mechanism of these processes remains ambiguous and existing findings are contradictory. Our results demonstrated that inhibition of mTORC1 for 1–48 h leads to suppression of metabolic activity and NSC proliferation. The effects of this inhibition could be detected even with a 48-h delay. Prolonged (24–48 h) inhibition of mTORC1 also led to inhibition of the mTORC2 complex, which in turn, alter the cytoskeletal proteins and cellular integrity. Furthermore, blocking the mTOR signaling pathway led to the suppression of neuronal differentiation from NSCs *in vitro*.

## Data availability statement

The original contributions presented in the study are included in the article/Supplementary material. The cell line iPSC-NP present in this study was obtained from Brigitte Onteniente, who provided it within the European project STEMS. Recently, the cell line is available as PCi-NPC from company Phenocell (Grasse, France). Further inquiries can be directed to the corresponding authors.

## Ethics statement

Ethical approval was not required for the studies on humans in accordance with the local legislation and institutional requirements because only commercially available established cell lines were used.

## Author contributions

NR: Data curation, Formal analysis, Investigation, Methodology, Supervision, Writing – original draft, Writing – review & editing. KS: Data curation, Investigation, Methodology, Writing – original draft. IA: Data curation, Writing – original draft. MH: Writing – review & editing. CG: Conceptualization, Validation, Writing – review & editing, Funding acquisition. MJ-U: Conceptualization, Supervision, Validation, Writing – review & editing. PJ: Conceptualization, Funding acquisition, Supervision, Validation, Writing – review & editing.
