# Supplementary material for: mTOR pathway inhibition alters proliferation as well as differentiation of neural stem cells
Source: Front Cell Neurosci. 2024 May 15;18:1298182. doi: 10.3389/fncel.2024.1298182 (PMC11133533; doi:10.3389/fncel.2024.1298182)
Supplement: Supplementary file 1 [file Table_1.DOCX]

**Table S1.** **List of antibodies used for immunofluorescence staining and for WB.** All antibodies are from Cell Signaling Technology, Danvers, MA, USA.

| Antibody | Western blot | ICC | Molecular weight (kDa) | Catalog number |
| --- | --- | --- | --- | --- |
| mTOR | 1:1000, BSA | 1:400 | 289 | 2983 |
| pmTOR^Ser2448^ | 1:1000, BSA | 1:400 | 289 | 5536 |
| MAP2 | 1:1000, BSA | 1:50 | 75,82,280 | 4542 |
| NF-H | 1:1000, milk | 1:400 | 180-220 | 2836 |
| nestin | 1:2000, BSA | 1:200 | 200 | 33475 |
| β1integrin | 1:1000, BSA | 1:400 | 115, 135 | 34971 |
| vinculin | 1:1000, BSA | x | 124 | 13901 |
| ALDH1L1 | 1:1000, BSA | 1:50 | 98 | 85828 |
| Akt | 1:1000, BSA | 1:200 | 60 | 9272 |
| pAkt^ser473^ | 1:1000, BSA | 1:400 | 60 | 4060 |
| βIII-tubulin | 1:1000, BSA | 1:400 | 55 | 5568 |
| NeuN | 1:1000, BSA | 1:50 | 46-55 | 24307 |
| GFAP | 1:1000, milk | 1:200 | 50 | 12389 |
| β-actin (HRP Conjugate) | 1:2000, TBST | x | 45 | 12620 |
| S6K1 | 1:1000, milk | 1:100 | 32 | 2317 |
| pS6K1^Ser235/236^ | 1:1000, BSA | 1:150 | 32 | 4858 |
